# METTL3-mediated m^6^A RNA methylation regulates dorsal lingual epithelium homeostasis

**DOI:** 10.1038/s41368-022-00176-2

**Published:** 2022-05-17

**Authors:** Qiuchan Xiong, Caojie Liu, Xin Zheng, Xinyi Zhou, Kexin Lei, Xiaohan Zhang, Qian Wang, Weimin Lin, Ruizhan Tong, Ruoshi Xu, Quan Yuan

**Affiliations:** 1 State Key Laboratory of Oral Diseases & National Clinical Research Center for Oral Diseases & West China Hospital of Stomatology, Sichuan University, Chengdu, China; 2 Department of Thoracic Oncology, Cancer Center, and Laboratory of Clinical Cell Therapy, West China Hospital, Sichuan University, Chengdu, China

**Keywords:** Stem-cell niche, Genetics research

## Abstract

The dorsal lingual epithelium, which is composed of taste buds and keratinocytes differentiated from K14^+^ basal cells, discriminates taste compounds and maintains the epithelial barrier. N6-methyladenosine (m^6^A) is the most abundant mRNA modification in eukaryotic cells. How METTL3-mediated m^6^A modification regulates K14^+^ basal cell fate during dorsal lingual epithelium formation and regeneration remains unclear. Here we show knockout of *Mettl3* in K14^+^ cells reduced the taste buds and enhanced keratinocytes. Deletion of *Mettl3* led to increased basal cell proliferation and decreased cell division in taste buds. Conditional *Mettl3* knock-in mice showed little impact on taste buds or keratinization, but displayed increased proliferation of cells around taste buds in a protective manner during post-irradiation recovery. Mechanically, we revealed that the most frequent m^6^A modifications were enriched in Hippo and Wnt signaling, and specific peaks were observed near the stop codons of *Lats1* and *FZD7*. Our study elucidates that METTL3 is essential for taste bud formation and could promote the quantity recovery of taste bud after radiation.

## Introduction

The lingual epithelium consists of non-taste epithelium and taste epithelium. The non-taste epithelium covers a large proportion of the tongue’s surface. In mice, fungiform papillae (FFP), surrounded by mechanosensory filiform papillae (FLP), are distributed in the front part of the dorsal lingual epithelium^1^. The single circumvallate papilla (CVP) in mice, containing numerous taste buds, is located in the midline of the posterior lingual epithelium, whereas singular FFP houses only one taste bud^2^.

The taste system is mediated by the taste buds and innervated sensory neurons. Murine taste buds contain 50–100 elongated epithelial cells, which can be categorized into several types (types I, type II, and type III)^3^. Through different specific receptors, taste buds can detect five taste qualities: bitter, salt, sweet, sour, and umami (savory)^4–6^. Dysgeusia is common in patients undergoing head and neck radiotherapy^7–9^. Some patients may recover from taste dysfunction after some months or years, but a small minority of patients may suffer from permanent taste loss^10,11^. Loss of taste buds after radiation is caused by natural taste cell death and the interruption of taste cell replenishment^8^.

Previous studies using lineage tracing indicate that basal cells expressing cytokeratin 5 (K5 or Krt5) and K14 are progenitors of both non-taste epithelium and taste epithelium in mice^12^. Taste buds undergo continuous turnover, with an average life span of 10–14 days, while the non-taste epithelium takes 5–7 days to be renewed^13,14^. In non-taste epithelium, K5/K14^+^ basal progenitors differentiate into K13^+^ (KRT13) keratinocytes, which make up the suprabasal epithelial layers of the FLP and FFP^15,16^. K5/K14^+^ basal progenitors can also generate new cells into taste buds, subsequently developing into mature taste cell types^1^. Type I cells resemble glia and are the most abundant cells present in taste buds. However, the specific function of type I cells remains elusive^17,18^. Some researchers regard type I cells as salt detector^4^. Type II cells can transduce different signals by detecting bitter, sweet, and umami tasting stimuli, whereas type III cells can transduce sour flavors^19–21^. These three different cell types can be identified via distinctive markers: type I cells express ecto-ATPase, NTPdase2; type II cells express α-gustducin, phospholipase Cβ2; and type III cells express NCAM and SNAP-25^22–24^.

As the most abundant mRNA modification, N6-methyladenosine (m^6^A) regulates mRNA fate, including stability, splicing, transport, localization, and translation^25–28^. Importantly, the m^6^A modification is a reversible and dynamic process. Recent studies have demonstrated that the m^6^A modification can be catalyzed by an RNA methyltransferase complex, which consists of methyltransferase-like 3 (METTL3), Wilms tumor 1-associated protein (WTAP), METTL14, and other proteins. This modification can be removed by m^6^A eraser proteins, including alkylation repair homolog protein 5 (ALKBH5) and fat mass and obesity-associated protein (FTO)^29–33^. Over the past few years, METTL3, the most important component of the RNA methyltransferase complex, has been reported to play critical functions in embryonic development, stem cell differentiation, and tumor progression^28,34–39^. However, the role of m^6^A modification in lingual epithelial homeostasis remains elusive.

Here, our group generated an epidermis-specific *Mettl3* knockout mouse model and found that METTL3 was an essential RNA methyltransferase that regulated lingual epithelium progenitor differentiation and was crucial for taste bud development. Moreover, overexpression of *Mettl3* promoted taste bud recovery from radiation injury. We also discovered a mechanistic pathway by identifying downstream target genes and signals.

## Results

### Deletion of *Mettl3* in epidermal progenitors leads to taste bud defects

**Fig. 1:**
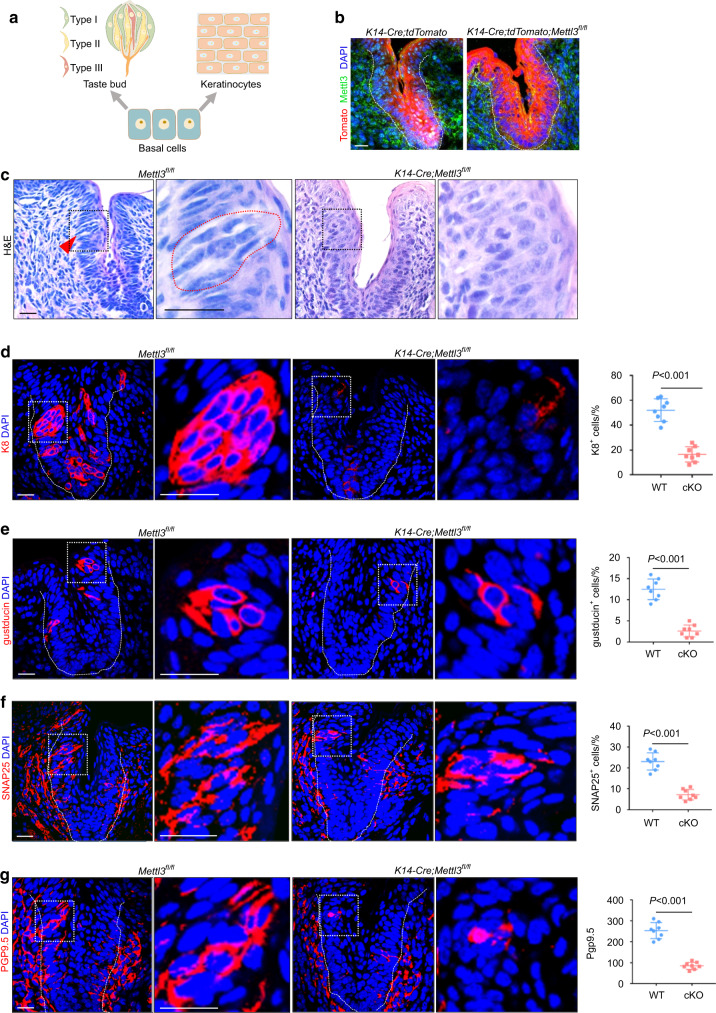
Deletion of *Mettl3* in epidermal progenitors leads to taste bud defects. **a** A schematic of basal progenitor cell differentiation. **b** METTL3 staining in circumvallate papillae from mice pups at P4. **c** H&E staining of taste buds (red arrow) in circumvallate papillae of P4 mice. **d** Staining and quantification of K8 + cells in circumvallate papillae (*n* = 8 biological replicates, *P* < 0.001 by unpaired two-tailed Student’s *t*-test). **e**, **f** Expression of GNAT3 and SNAP25 and the indicated quantification (*n* = 8 biological replicates, *P* < 0.001 by unpaired two-tailed Student’s *t*-test). **g** Detection of PGP9.5 in circumvallate papillae. Integrated density of PGP9.5 per trench profile (*n* = 8 biological replicates, *P* < 0.001 by unpaired two-tailed Student’s *t*-test). Dotted lines in **b, d, e, f**, and **g** indicate basal lamina. Scale bar: 20 μmol·L^−1^

As previously described, basal progenitors develop into taste cells (K8) and differentiated keratinocytes (K13) (Fig. 1a)^15,16,40^. To explore whether deletion of *Mettl3* affected taste bud development, we crossed *Mettl3*^*fl/fl*^ mice^28^ with *K14-Cre* transgenic mice to conditionally delete *Mettl3* from epithelium basal progenitors. *K14-Cre;Mettl3*^*fl/fl*^ mice were viable and born at Mendelian’s ratio. Most of them survived by postnatal day 4 (P4), and few could survive by P7. We also generated the *K14-Cre;tdTomato;Mettl3*^*fl/fl*^ mice, in which K14^+^ cells and their daughter cells were labeled with tdTomato fluorescence, and confirmed that METTL3 was largely abolished within Tomato^+^ cells in the CVP (Fig. 1b).

Because of their short lifespan, we sacrificed *K14-Cre; Mettl3*^*fl/fl*^ mice at P4 to explore whether taste bud differentiation was affected. Compared to control CVPs, the taste buds in mutant CVPs could not be recognized by hematoxylin and eosin (H&E) staining (Fig. 1c). K8 is a marker of differentiated taste bud cells^40^. Immunofluorescence staining revealed that the number of K8^+^ cells in the CVP was significantly decreased (Fig. 1d). We also observed a reduced number of type II cells (marked by gustducin) and type III cells (marked by SNAP25) in mutant CVP (Fig. 1e, f). Consistent with the taste bud loss phenotype, the innervated areas (marked by PGP9.5) of mutant CVP were remarkably reduced (Fig. 1g).

### Deletion of *Mettl3* leads to abnormal keratinization of lingual epithelium

**Fig. 2:**
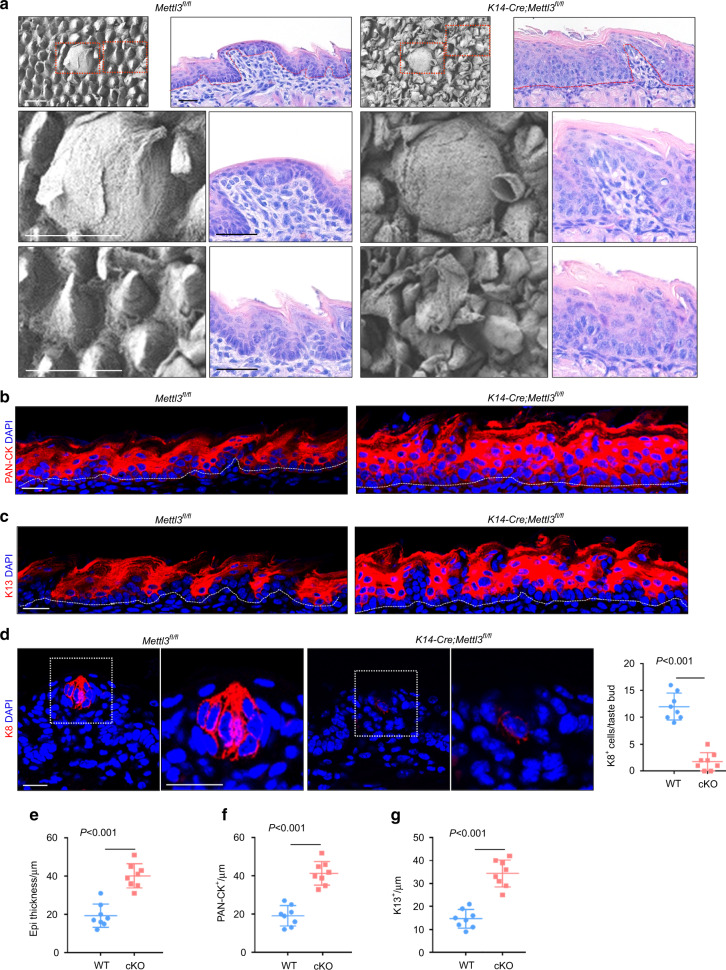
Deletion of *Mettl3* leads to abnormal keratinization of lingual epithelium. **a** P4 *K14-cre; Mettl3*^*fl/fl*^ mice displayed abnormal filiform papilla and keratinized fragments (red arrow), assayed by SEM. Scale bar: 50 μmol·L^−1^. Deletion of *Mettl3* caused the epithelium thickening, as revealed by H&E staining. Scale bar: 20 μmol·L^−1^. **b** The detection of PAN-CK indicates epithelial thickening in mutant mice. Scale bar: 20 μmol·L^−1^. **c** Increased expression of keratinocyte markers K13 in *K14-cre; Mettl3*^*fl/fl*^ mice. Scale bar: 20 μmol·L^−1^. **d** Decreased expression of taste bud markers K8 and quantification of K8 + cells in *K14-cre; Mettl3*^*fl/fl*^ fungiform papillae section (for each condition *n* = 8 biological replicates, *P* < 0.001 by unpaired two-tailed Student’s *t*-test). Scale bar: 20 μmol·L^−1^. Dotted lines in **a**, **b**, and **c** indicate the basal lamina. **e** Quantitative analysis of entire epithelium thickness, *P* < 0.001 by unpaired two-tailed Student’s *t*-test. **f** Quantitative analysis of PAN-CK^+^ epithelium thickness, *P* < 0.001 by unpaired two-tailed Student’s *t*-test. **g** Quantitative analysis of K13^+^ epithelium thickness, *P* < 0.001 by unpaired two-tailed Student’s *t*-test)

We then examined whether METTL3 regulated keratinization of the lingual epithelium. Scanning electron microscopy (SEM) showed that epithelial-specific *Mettl3* deletion caused morphological abnormalities in FLP at P4 (Fig. 2a). Excessive keratinized fragments were observed on the surface of the tongue epithelium in mutant mice (Fig. 2a). H&E staining showed that the thickness of the epithelium was increased and the cell alignment was irregular compared to that of control mice (Fig. 2a). Quantitative analysis of epithelium thickness showed that the entire epithelium thickness of the mutants was almost double that of the controls (Fig. 2e). This observation was confirmed by immunofluorescence staining of PAN-CK and K13 cells (Fig. 2b, c, f, g). Although there was no obvious difference in the appearance of FFP by SEM, the number of K8^+^ taste cells in FFP decreased in the mutant mice (Fig. 2d).

### Deletion of *Mettl3* increases basal cell proliferation but decreases cell proliferation around taste buds

**Fig. 3:**
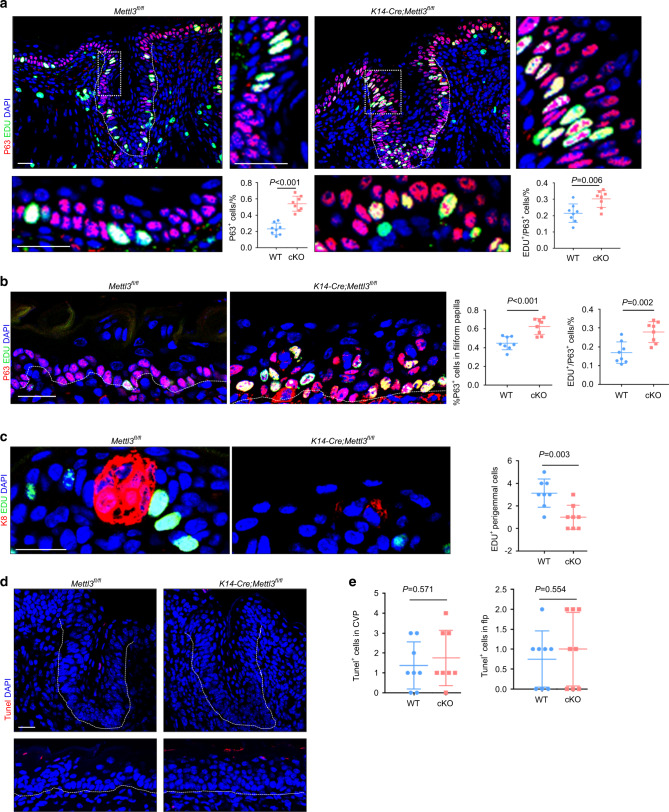
Deletion of *Mettl3* increases basal cell proliferation but decreases cell proliferation around taste buds. **a** Immunofluorescence for P63 and EdU in (**a**) Immunofluorescence for P63 and EdU in circumvallate papillae. Scale bar: 20 μmol·L^−1^. Quantification results are shown (for each condition n = 8 biological replicates, *P* < 0.001 and *P* = 0.006 by unpaired two-tailed Student’s *t*-test). **b** Immunofluorescence staining for P63 and EdU in filiform papillae. P63 + cells/total cells and EdU+ cells/P63 + cells are counted (for each condition *n* = 8 biological replicates, *P* < 0.001 and *P* = 0.002 by unpaired two-tailed Student’s *t*-test). **c** Immunofluorescence staining for K8 and EdU of perigemmal cells in fungiform papillae. Scale bar: 20 μmol·L^−1^. EDU + perigemmal cells are counted in fungiform papillae (for each condition *n* = 8 biological replicates, *P* = 0.003 by unpaired two-tailed Student’s *t*-test). **d** There is no difference in apoptosis, as assayed by TUNEL immunofluorescence. Scale bar: 20 μmol·L^−1^. **e** Quantitative analysis of TUNEL^+^ cells in CVP, *P* = 0.571 by unpaired two-tailed Student’s *t*-test). Quantitative analysis of TUNEL^+^ cells in FLP, *P* = 0.554 by unpaired two-tailed Student’s *t*-test)

Transcription factor p63 is a crucial regulator of epidermal development and marks basal stem cells^41^. We noted that the number of P63^+^ cells in the CVP increased in the mutants (Fig. 3a). According to immunofluorescence results for P63 and 5-ethynyl-2′-deoxyuridine (EdU), the proliferation of P63^+^ cells in mutant CVP was more active than in control mice (Fig. 3a). Consistent results were observed in the FLP analysis (Fig. 3b). Deletion of *Mettl3* led to an increase in the number of P63^+^ cells and promoted the proliferation of basal cells (Fig. 3b). In contrast, cell proliferation around taste buds reduced in *Mettl3* knockout mice (Fig. 3c). Deletion of *Mettl3* did not affect apoptosis of taste or non-taste cells (Fig. 3d, e).

### Overexpression of *Mettl3* promotes taste bud recovery after irradiation

**Fig. 4:**
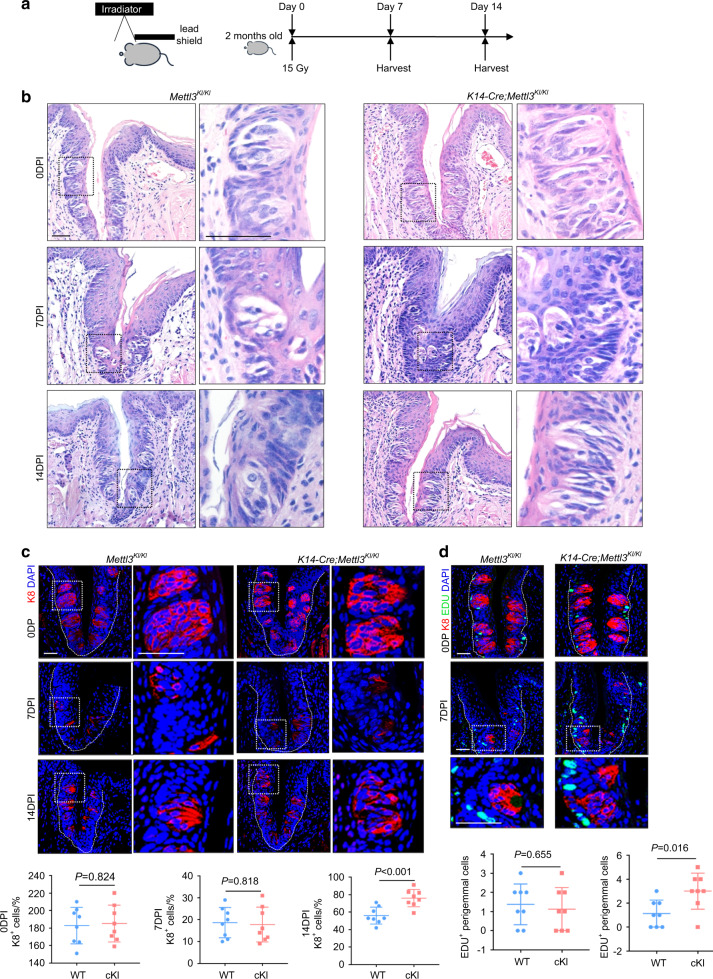
Overexpression of *Mettl3* promotes taste bud recovery after irradiation. **a** Injury model for irradiation. **b** H&E-stained circumvallate papillae sections reveal that overexpression of *Mettl3* does not impact taste bud development in normal physiology (0 dpi), nor protect taste buds from irradiation (7 dpi). Taste buds in mutants are significantly increased by 14 dpi. Scale bar: 20 μmol·L^−1^. **c** Immunofluorescence of K8 shows taste buds at 0 dpi, 7 dpi and 14 dpi. Scale bar: 20 μmol·L^−1^. K8 + cells in circumvallate papillae are counted (for each condition *n* = 8 biological replicates, *P* = 0.824 (0 dpi), *P* = 0.818 (7 dpi), *P* < 0.001 (14 dpi) by unpaired two-tailed Student’s *t*-test). **d** Immunofluorescence staining for K8 and EdU reveals increased proliferation of perigemmal cells in circumvallate papillae. Scale bar: 20 μmol·L^−1^. EDU + perigemmal cells are counted in circumvallate papillae (for each condition *n* = 8 biological replicates, *P* = 0.665 (0 dpi), *P* = 0.016 (7 dpi) by unpaired two-tailed Student’s *t*-test)

Next, we investigated whether *Mettl3* overexpression could prevent lingual epithelial disorders. To this end, we generated *K14-Cre* driven *Mettl3-tdTomato* knock-in mice (*K14-Cre;Mettl3*^*KI/KI*^) to conditionally overexpress *Mettl3* in epidermal progenitor cells^28^. As *K14-Cre;Mettl3*^*KI/KI*^ mice did not exhibit a significant change in taste bud and epithelium development, we challenged them with 15 Gy irradiation (Fig. 4a). Both knock-in and control mice exhibited a severe loss of taste buds at 7 days post-irradiation (dpi) (Fig. 4b, c), indicating that overexpression of *Mettl3* could not protect mice from epithelial injury due to irradiation. Notably, at 14 dpi, there were more recovered taste buds in the knock-in mice (Fig. 4b, c). Furthermore, overexpression of *Mettl3* increased the proliferation of cells around taste buds at 7 dpi (Fig. 4d).

### METTL3-mediated m^6^A RNA methylation regulates Hippo and Wnt pathways

**Fig. 5:**
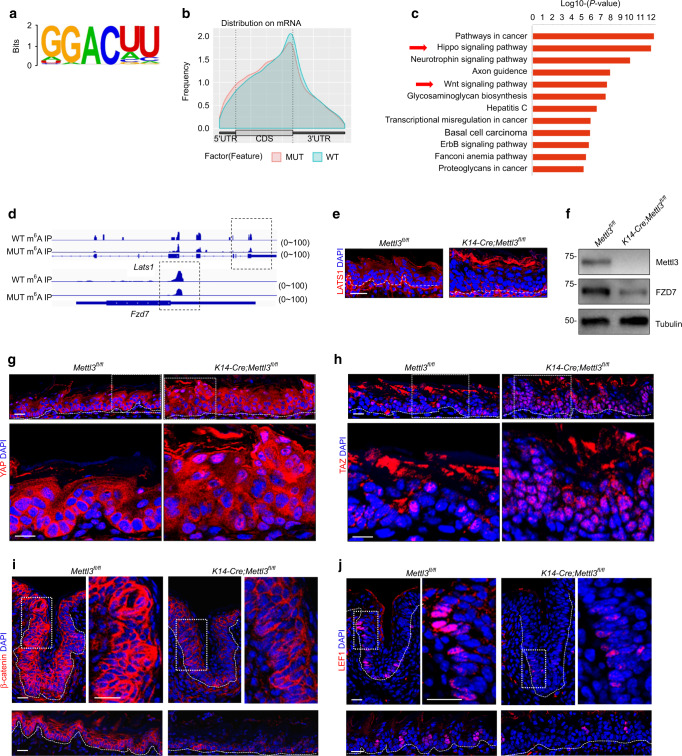
METTL3-mediated m^6^A RNA methylation regulates Hippo and Wnt pathways. **a** Consensus motif of m^6^A sites in *Mettl3*^*fl/fl*^ mice. **b** Enrichment of m6ARIP-seq peaks in *Mettl3*^*fl/fl*^ mice and *K14-cre; Mettl3*^*fl/fl*^ mice at P4. **c** KEGG pathway enrichment for highly modified mRNAs. **d** The read density from m6ARIP-seq experiments shows the m^6^A peaks in *Mettl3*^*fl/fl*^ mice and decreased peaks in *K14-cre; Mettl3*^*fl/fl*^ mice. **e** Decreased expression of LATS1 in conditional *Mettl3* knock-out mice at P4. Scale bar: 20 μmol·L^−1^. **f** Western blot analysis of FZD7 in *Mettl3*^*fl/fl*^ mice and *K14-cre; Mettl3*^*fl/fl*^ mice. **g**, **h** Immunofluorescence assays are used to detect the expression and location of YAP and TAZ. Scale bar: 20 μmol·L^−1^. **i, j** Immunofluorescence assays are used to detect the expression and localization of β-catenin and LEF1. Scale bar: 20 μmol·L^−1^

To explore the underlying mechanisms, we performed m^6^A RNA immunoprecipitation sequencing (m^6^ARIP-seq) of the lingual epithelium in *K14-Cre; Mettl3*^*fl/fl*^ mice and their *Mettl3*^*fl/fl*^ littermates at P4. Consistent with previous reports^28,42^, m^6^A peaks shared a common GGACU motif (Fig. 5a) and were enriched around the stop codon (Fig. 5b). Gene pathway analysis revealed that the most frequent changes involving m^6^A modifications after *Mettl3* deletion were enriched in the Hippo and Wnt signaling pathways (Fig. 5c).

Specific peaks were observed near the stop codons of the *large tumor suppressor kinase 1 (Lats1)* and *Frizzled class receptor 7 (Fzd7)* (Fig. 5d). The deletion of *Mettl3* significantly decreased the abundance of m^6^A modifications (Fig. 5d). Immunofluorescence staining and western blot analysis confirmed reduced LATS1 and FZD7 protein levels, respectively (Fig. 5e, f). Loss of METTL3 led to increased nuclear localization of YAP and TAZ in the basal epithelium (Fig. 5g, h). FZD7, a Wnt receptor, transduces signals and activates the Wnt signaling pathway^43,44^. Immunofluorescence staining for β-catenin indicated that nuclear β-catenin expression by *K14-Cre; Mettl3*^*fl/fl*^ mice at P4 was significantly decreased in terms of both taste and non-taste epithelia (Fig. 5i). In addition, LEF1 expression was decreased in *K14-Cre; Mettl3*^*fl/fl*^ mice (Fig. 5j).

## Discussion

In recent years, many studies have reported a role for METTL3 in stem cell differentiation. METTL3-mediated m^6^A modification regulates the development of the hematopoietic system, spermatogenesis, and other organs^45,46^. Our research group also found that m^6^A modification mediated by METTL3 plays a key role in regulating the fate of bone marrow mesenchymal stem cells^28^. In this study, we uncovered an essential role for METTL3 in lingual epithelial homeostasis. METTL3 was widely expressed in the lingual epithelium. Deletion of *Mettl3* led to severe defects in taste bud development and abnormal epithelial thickening. In addition, overexpression of *Mettl3* promoted taste bud recovery from radiation damage. Furthermore, the Wnt and Hippo signaling pathways may be responsible for the striking phenotypic defects in taste buds and keratinizing epithelium.

The Wnt/β-catenin pathway is required for taste bud development and taste cell renewal^15,47,48^. Basal cells with high expression of β-catenin can give rise to taste cells, whereas lower levels of β-catenin expression promote keratinocyte fate^1,15^. Conditional β-catenin knockout in progenitor cells causes a decrease in taste cells^48^. Here, we showed that the deletion of *Mettl3* resulted in the downregulation of β-catenin and LEF1, which led to defects in taste cell development.

However, a study demonstrated that conditional knockout of *Ctnnb1* in the epidermis led to FLP and FFP developmental defects and thinner epithelium, which did not match our phenotype^49^. In our study, β-catenin expression was extremely low after *Mettl3* knockout, whereas the lingual epithelium was thickened. This finding reminded us that different mechanisms regulate the non-taste epithelium. METTL3-mediated m^6^A modification has been identified as the most abundant mRNA modification that regulates biological processes in mRNA^27,50^. Deletion of *Mettl3* reduces the m^6^A peaks of many mRNAs, which changes their fate choices. The m^6^ARIP-seq analysis of the lingual epithelium showed that m^6^A modifications were mainly enriched in the Hippo and Wnt signaling pathways (Fig. 5c). LATS1 is a crucial kinase that phosphorylates and inactivates the transcriptional coactivators YAP and TAZ^51–53^. In our study, we found that loss of METTL3 reduced the m^6^A modification of LATS1 and further inhibited the Hippo pathway, resulting in abnormal proliferation of keratinizing epithelium.

Another area for investigation is how METTL3 regulates cellular physiological functions and pathological progression through m^6^A modification. A number of studies have elaborated on the role of RNA m^6^A modifications in alternative splicing^42,45^. A recent study also pointed out that m^6^A methylation regulates an array of chromosome-associated regulatory RNAs (carRNAs) to globally tune chromatin state and gene transcription^54^. In some studies, METTL3 and m^6^A modifications have been shown to enhance mRNA stability to promote cell proliferation^37,55^. m^6^A has been shown to promote mRNA translation in certain cell types^56,57^. In our previous study, the deletion of *Mettl3* slowed down the translation of target proteins and further inhibited downstream signaling^28^. m^6^A modifications are important and function depending on the cellular context. Thus, we did not further investigate mRNA metabolism in this study.

Dysgeusia is common and significant in patients after receiving head and neck radiotherapy. A previous study found that radiation interrupts the renewal of taste bud cells by inhibiting the proliferation and differentiation of basal progenitor cells, resulting in taste bud injury in mice^8^. In subsequent studies, they found that activation of the Wnt/β-catenin signaling pathway promotes the recovery of taste cells from radiation^58^. To determine the function of METTL3 in radiation-induced gustation dysfunction, conditional *Mettl3* knock-in mice were exposed to radiation and analyzed for taste bud maintenance and recovery. We found that overexpression of *Mettl3* promoted taste bud recovery from radiation damage by increasing the proliferation of taste bud progenitor cells. Interestingly, overexpression of *Mettl3* did not protect taste buds from radiation injury.

In conclusion, we elucidated that METTL3 was an essential regulator of lingual epithelial homeostasis by regulating m^6^A modification. Deletion of *Mettl3* in the epidermis reduced the expression of LATS1 and FZD7 and further blocked downstream pathways, which led to taste bud defects and epithelial thickening. In addition, overexpression of *Mettl3* promoted taste bud recovery from radiation damage by increasing the proliferation of taste bud progenitor cells.

## Materials and methods

### Mice

*Mettl3*^*fl/+*^ and *Mettl3*^*KI/+*^ mice were generated using the CRISPR-Cas9 system, as described previously^28^. *K14-Cre* transgenic mice were kindly provided by Dr. Demeng Chen from Sun Yat-sen University (Guangzhou, China). *Rosa26-tdTomato* mice were purchased from the Jackson Laboratory (Cat No:007905, Pennsylvania, USA). *Mettl3*^*fl/fl*^ and *Mettl3*^*KI/KI*^ mice were crossed with *K14-Cre* transgenic mice to generate *K14-Cre; Mettl3*^*fl/fl*^ mice and *K14-Cre; Mettl3*^*KI/KI*^ mice. By mating *Mettl3*^*fl/fl*^ mice with *K14-Cre; tdTomato* mice, we obtained *K14-Cre; tdTomato; Mettl3*^*fl/fl*^ mice. All mice had a C57BL6/J background. The genotypes of transgenic mice were identified as previously described^28^.

All mice were housed in specific pathogen-free (SPF) facilities with a 12-hour light-dark illumination cycle. All studies performed on mice were approved by the Subcommittee on Research and Animal Care (SRAC) at Sichuan University.

### Tissue preparation

After anesthesia with xylazine (10 mg·kg^−1^) and ketamine (80 mg·kg^−1^), mice were perfused transcardially with normal saline and 4% paraformaldehyde (PFA) in 0.1 mol·L^−1^ phosphate buffer. Tongues were dissected from the mandible and fixed in 4% PFA overnight at 4 °C. For frozen sections, tissues were transferred to 20% sucrose in 0.1 mol·L^−1^ phosphate buffer overnight at 4 °C. The samples were embedded in OCT compound (Sakura Finetek, Torrance, USA) and cryosectioned to 12 µm. For paraffin sections, the samples were dehydrated in graded ethanol and xylene and then embedded in 5-µm-thick paraffin sections using a microtome (Leica, RM2255, Wetzlar, German).

### Histology and immunofluorescence

For hematoxylin and eosin (H&E) staining, the paraffin sections were de-waxed using graded xylene solutions. Staining was performed according to the manufacturer’s instructions (Solarbio Science and Technology, Beijing, China).

For immunohistochemistry, paraffin sections were prepared by the above procedures, microwaved in sodium citrate buffer, and incubated with primary and secondary antibodies (Boster Biological Technology, Wuhan, China). Finally, the sections were developed using the AEC (3-amino-9-ethylcarbazole) Staining Kit (Boster Biological Technology, Wuhan, China).

For immunofluorescence, cryosections were thawed at room temperature (26 °C), rehydrated in 0.1 mol·L^−1^ phosphate-buffered saline and microwaved in sodium citrate buffer. After incubation with the primary antibody, the sections were incubated with Alexa Fluor-labeled secondary antibodies (Jackson Laboratory, Pennsylvania, USA).

### Antibodies

The following antibodies were used: rabbit anti-METTL3 (1:200; Abcam, Cat No: ab195352), rabbit anti-KRT8 (1:200; Abcam, Cat No: ab53280), goat anti- gustducin (1:100; Aviva Systems Biology, Cat No: OAEB00418), rabbit anti-PGP9.5 (1:500, Thermo Fisher, Cat No: 480012), rabbit anti-SNAP25 (1:100; Proteintech, Cat No: 14903-1-AP), mouse anti- PAN-CK(1:100, Thermo Fisher, Cat No: MA1-82041), mouse anti-KRT13 (1:200; Abcam, Cat No: ab16112), rabbit anti-p63 (1:300; Abcam, Cat No: ab53039), mouse anti-LATS1 (1:100; Santa Cruz Biotechnology, Cat No: sc-398560), mouse anti-YAP (1:200; Santa Cruz Biotechnology, Cat No: sc-101199), mouse anti-TAZ (1:100; Santa Cruz, Cat No: sc-293183), rabbit anti-FZD7 (1:1 000 for western blot; Abcam, Cat No: ab64636), rabbit anti-β-catenin (1:250; Proteintech, Cat No: 51067-2-AP), rabbit anti-LEF1 (1:200; Abcam, Cat No: ab137872).

### Irradiation

After anesthesia, the mice were covered with a custom-made lead shield, leaving the head and neck exposed. The mice were irradiated with 15 Gy in an X-ray irradiator (~1.25 Gy per min, Accela, X-RAD 160). Irradiated mice were returned to their cages for recovery.

### TUNEL assay

To assess cell death, the TUNEL assay was performed using the In Situ Cell Death Detection Kit TMR Red (Boster Biological Technology, MK 1012-100, Wuhan, China). The sections were digested with proteinase K for 5 min and then washed in tris-HCl buffered saline three times. Sections were incubated with labeling buffer for 2 h at 37 °C prior to TUNEL reactions. Labeling buffer was prepared according to the manufacturer’s instructions. After two washes, the sections were incubated in blocking solution at room temperature for 30 min. The blocking solution was then removed. The fluorescence probes were used to detect cell death. Sections were counterstained with DAPI and imaged using laser scanning confocal microscopy (LSCM; Olympus FV3000, Tokyo, Japan).

### EdU labeling

For EdU labeling, mice were injected with 25 µg of EdU per gram of body weight and euthanized after 1 h. Tongues were fixed overnight in 4% PFA and embedded in paraffin. After de-waxing, paraffin sections were incubated with the Click-iT EdU Imaging Kit (Invitrogen, CA, USA).

### SEM

Tongue samples were fixed in 4% PFA overnight and dehydrated in a graded series of ethanol concentrations. Dehydrated samples were then incubated in 50% hexamethyldisilazane (Sigma-Aldrich, St. Louis, USA) for 20 min, followed by three solvent changes to 100% hexamethyldisilazane. After air-drying overnight, the samples were sputter-coated with gold-palladium. Specimens were examined and photographed using a SEM^49^.

### Western blot

The lingual epithelium was collected from four mice in each group following previously described methods^13^. The tissues were lysed in RIPA buffer (Pierce, Rockford, IL, USA). The sample was then mixed with sample buffer containing 2% SDS and 1% 2-mercaptoethanol and heated at 95 °C for 5 min. The samples were separated on 10% SDS–polyacrylamide gels and transferred to polyvinylidene fluoride (PVDF) membranes using a semi-dry transfer apparatus (Bio-Rad, CA, USA)^59^. Incubation with rabbit anti-METTL3 (1:2 000; Abcam, Cat No: ab195352), rabbit anti-FZD7 (1:1 000; Abcam, Cat No: ab64636), rabbit anti-α-tubulin (1:2 000; Proteintech, Cat No: 11224-1-AP) at 4 °C was conducted overnight, followed by incubation with a horseradish peroxidase (HRP)-conjugated secondary antibody (1:5 000, Cell Signaling Technology, Cat No:7074). After washing, the blots were analyzed with Immobilon Reagents (Millipore, Burlington, USA) using a gel imaging system (Bio-Rad, CA, USA).

### m^6^A MeRIP-Seq (m^6^A RNA immunoprecipitation sequencing)

Total RNA from the lingual epithelium was isolated using TRIzol reagent^13^. mRNA was enriched from total RNA using Immobilon Reagents (Millipore, Burlington, USA) and gel imaging systems (Bio-Rad, CA, USA). The mRNA was then fragmented with ZnCl_2_ buffer. Immunoprecipitation was performed using an anti-m^6^A antibody (1:100; Synaptic Systems, Cat No: 202003). Immunoprecipitated RNA was analyzed by high-throughput sequencing or RT-qPCR, as previously described^28,60^.

### Statistical analysis

All data are presented as means ± standard error. For comparison between two independent groups, statistical differences were analyzed using unpaired two-tailed Student’s *t*-test. Statistical significance was set at *p* < 0.05.

## Data Availability

All data are available in the main text.
